# The distribution of neonicotinoids and their metabolites in paired semen and urine samples and associations with male reproductive hormones

**DOI:** 10.3389/fendo.2026.1829662

**Published:** 2026-08-12

**Authors:** Lu Zhang, Li-Jun Lei, Zi-Jun Zhou, Qing-Shuang Yang, Fang-Di Dong, Zhi-Wei Xue, Jiao-Jing Liu, Ling-Yuan Wu, Zhi-Peng Cheng, Yu-Bin Ding, Hui-Jun Yang

**Affiliations:** 1 Assisted Reproductive Center, Key Laboratory of Birth Regulation and Control Technology of National Health Commission of China, Shandong Provincial Key Medical and Health Discipline of Reproductive Medicine, Shandong Provincial Maternal and Child Health Care Hospital Affiliated to Qingdao University, Jinan, China; 2 Joint International Research Laboratory of Reproduction and Development of the Ministry of Education of China, School of Public Health, Chongqing Medical University, Chongqing, China; 3 MOE Key Laboratory of Pollution Processes and Environmental Criteria, College of Environmental Science and Engineering, Nankai University, Tianjin, China; 4 Department of Pharmacology, Academician Workstation, Changsha Medical University, Changsha, China

**Keywords:** human biomonitoring, neonicotinoid insecticides, reproductive hormone, semen, urine

## Abstract

**Background:**

Neonicotinoids (NEOs) are widely used pesticides with potential endocrine-disrupting properties, yet their distribution in human reproductive matrices and associations with male reproductive hormones remain unclear.

**Methods:**

We analyzed 146 paired semen and urine samples to characterize the distribution of 14 NEOs and their metabolites (m-NEOs) and to examine associations with male reproductive hormones. Concentrations were compared across matrices, and associations with follicle-stimulating hormone (FSH), luteinizing hormone (LH), testosterone (T), prolactin (PRL), estradiol (E_2_), and the T/LH ratio were evaluated using exposome-wide association (EWAS), deletion–substitution–addition (DSA), and mixture models.

**Results:**

Detection frequencies ranged from 43.2% to 100% in urine and 19.9% to 99.3% in semen, with Desnitro-dinotefuran (DN) and Desmethyl-acetamiprid (DM-ACE) predominant in urine and DN and Acetamiprid (ACE) enriched in semen. Partitioning analysis revealed three distinct compound clusters: (1) DN, ACE, and Imidacloprid-urea (IMI-urea) enriched in semen; (2) Dinotefuran-urea (UF), Imidacloprid (IMI), Imidaclothiz (IMIT), and 5-Hydroxy-imidacloprid (5-OH-IMI); and (3) DM-ACE, Desnitro-imidacloprid (DN-IMI), Desnitro-imidacloprid-olefin (DN-IMI-olefin), and Clothianidin (CLO) predominantly excreted in urine. In EWAS and DSA, several nominal associations with hormones were observed. Nominal associations were observed between seminal CLO and FSH, DN and E_2_, and urinary UF and LH, Thiacloprid (THCP) exposure showed nominal positive associations with PRL and E_2_. However, none remained significant after correction for multiple testing. Mixture models indicated no overall significant associations. Sensitivity analyses supported the robustness of key compound–hormone associations.

**Conclusion:**

NEOs and m-NEOs show distinct partitioning patterns between urine and semen, suggesting differential tissue distribution. Although several nominal associations with reproductive hormones were identified, none remained statistically significant after multiple-testing correction, indicating that these findings should be considered hypothesis-generating rather than conclusive. Further studies with larger populations and repeated measurements are needed to validate these associations and clarify the reproductive implications of NEO exposure in men.

## 1 Introduction

Neonicotinoids (NEOs) are extensively applied in modern agriculture due to their high efficacy and selectivity against insect pests (1). These compounds have been detected in a wide range of environmental matrices, including soils (2), surface waters (3), air (4), indoor dust (5), and various foods (6) such as vegetables, fruits, and honey. Biomonitoring surveys further confirm human exposure, as NEOs and their metabolites have been detected in urine, serum, breast milk, saliva, and hair (5, 7–9), reflecting ubiquitous and continuous internal exposure in the general population.

Although primarily designed to target insect nicotinic acetylcholine receptors, NEOs are not biologically inert in humans. Beyond their neurotoxicity in insects, NEOs have emerged as potential endocrine-disrupting chemicals. Experimental studies show that NEOs and their metabolites impair reproductive and hormonal regulation, with animal models reporting reduced epididymis and seminal vesicle weights (10), increased sperm apoptosis and DNA fragmentation (11), and suppressed testicular function (12). Mechanistic findings also point to NEO-induced oxidative stress, mitochondrial dysfunction, and steroidogenic disruption as potential pathways of toxicity (13). Due to these findings in laboratory animals, there is growing public concern that ubiquitous exposure to NEOs may affect human male reproductive health.

Epidemiological findings remain inconsistent. Urinary NEOs have been associated with altered steroid hormone profiles and oxidative stress biomarkers (14), but reported associations with reproductive hormones vary across populations. For instance, in male farmworkers, urinary imidacloprid (IMI) and its metabolite Of-IMI were positively associated with testosterone (15), whereas NHANES data suggested decreased serum testosterone levels with higher NEO exposure (16). Other studies reported null associations (17), and research in Ecuadorian adolescents from agricultural communities observed positive associations between urinary NEOs and 17β-estradiol levels (17, 18). Collectively, these inconsistent results underscore the need for more refined exposure assessment approaches.

Most existing studies rely on urine or blood, which reflect recent or systemic exposure but may not capture burdens at reproductive target organs (19). Semen, in contrast, is a biologically relevant fluid that directly represents the testicular and accessory gland environment. Recent non-targeted and targeted analyses have identified hundreds of organic contaminants in seminal plasma, with chemical compositions and ratios distinct from those in blood and urine, and even demonstrated that some compounds (e.g., triclosan) can cross the blood–testis barrier and become enriched in seminal plasma (20, 21). Consistently, a growing body of evidence indicates that diverse environmental contaminants—including endocrine-disrupting chemicals such as phthalate metabolites (22), per- and polyfluoroalkyl substances (23), as well as trace metals like vanadium (24)—can be detected in semen and are associated with impaired male reproductive function. For NEOs, however, only one study has detected multiple metabolites in seminal plasma and suggested that IMI-olefin was inversely associated with semen quality (25), leaving their internal distribution and hormonal relevance largely unexplored.

Recent studies have also advanced our understanding of neonicotinoid metabolism and disposition in humans. Harada et al. (26) reported urinary excretion profiles and excretion kinetics of IMI and its major metabolites in humans, providing *in vivo* evidence for the occurrence of 5-hydroxy-IMI, Imidacloprid-urea (IMI-urea), and desnitro-IMI (DN-IMI) in human biological samples, while Wrobel et al. (27, 28) identified N-desmethyl-acetamiprid (DM-ACE) as a human-relevant metabolite of acetamiprid (ACE). These findings suggest that not all neonicotinoid-related compounds found in biological samples are necessarily formed *in vivo*—some may arise from environmental degradation or direct exposure to transformation products.

To fill this gap, we conducted a population-based biomonitoring study in adult men, simultaneously measuring a broad panel of NEOs and their metabolites in paired urine and semen samples. We systematically characterized detection frequencies, concentration distributions, and partitioning patterns between matrices, and further examined associations with male reproductive hormones. This paired-matrix design provides the first comprehensive evidence of NEO distribution within reproductive target fluids and its potential endocrine implications, offering novel insights into the internal kinetics of pesticide exposure and male reproductive health.

## 2 Materials and methods

### 2.1 Chemicals and reagents

Analytical standards of parent neonicotinoids (NEOs; ACE, IMI, Clothianidin (CLO), Thiacloprid (THCP), Thiamethoxam (THM), and Dinotefuran(DNT)) and their metabolites (m-NEOs; FLO, Imidaclothiz (IMIT), Descyano-ACE, DM-ACE, DN-IMI, DN-IMI-olefin, IMI-urea, 5-Hydroxy-IMI, THCP-amide, Desmethyl-thiamethoxam (DM-THM), Desnitro-dinotefuran (DN), and Dinotefuran-urea (UF)) were purchased from Sigma-Aldrich (St. Louis, MO, USA) and Dr. Ehrenstorfer GmbH (Augsburg, Germany).The isotope-labeled internal standards (ISs)—Acetamiprid-*d_3_* (ACE-*d_3_*), Clothianidin-*d_3_* (CLO-*d_3_*), Imidacloprid-*d_4_* (IMI-*d_4_*), Thiacloprid-(thiazolidin-ring-*d_4_*) (THCP-*d_4_*), and Thiamethoxam-*d_4_* (THM-*d_4_*)—were provided by Prof. Tao Zhang’s group at Sun Yat-sen University (Guangzhou, China). Comprehensive details on the full names, abbreviations, CAS numbers, formulas, molecular weights, and structure of the 18 NEOs and 5 ISs are presented in **Supplementary Table 1**.

Individual stock solutions of unlabeled analytes were prepared in HPLC-grade methanol at concentrations ranging from 10 to 1000 ppm depending on solubility. Specifically, single-standard solutions of m-NEOs were prepared at 1000 ppm (0.01 mL in 4.93 mL methanol), while mixed standard solutions of parent NEOs and certain metabolites were prepared at 10 ppm. A mixed external standard working solution (1 ppm, 10 mL) was obtained by combining appropriate aliquots of these stock solutions and diluting with methanol. The internal standard working solution (1 ppm, 10 mL) was prepared by mixing 0.1 mL of DNT-*d_3_* (100 ppm) and 1 mL of the 10 ppm IS mixture (ACE-*d_3_*, CLO-*d_3_*, IMI-*d_4_*, THCP-*d_4_*, and THM-*d_4_*), followed by dilution with 8.9 mL of methanol (HPLC-grade). All standards were stored in amber vials at −20°C before use.

Formic acid (>95%), acetic acid (>99%), and β-glucuronidase were obtained from Sigma-Aldrich. Methanol (HPLC-grade) and ethyl acetate (>99%) were purchased from Fluka and J.T. Baker (USA), respectively. Ultrapure water was generated using a Milli-Q purification system (Barnstead International, Dubuque, IA, USA). The purity of all standards was >97%.

### 2.2 Study population and sample collection

The study population consisted of male partners of infertile couples seeking assisted reproductive technology (ART) services at a hospital in Shandong Province. The indications for ART included male-factor infertility, female-factor infertility, combined infertility factors, and unexplained infertility. Therefore, the enrolled participants represented a heterogeneous group of men with varying reproductive health statuses rather than exclusively men with male-factor infertility. Urine and semen samples were collected from March to November 2024 during routine clinical visits. Eligible participants were men aged over 20 years without a history of severe systemic or hereditary diseases. No upper age limit was prespecified. Exclusion criteria aimed to minimize confounding factors that could profoundly and uncontrollably alter sex hormone levels. Specifically, patients were excluded if they met any of the following conditions: (1) diagnosed major endocrine disorders such as pituitary adenoma, hyperprolactinemia, or diabetes mellitus; presence of chromosomal abnormalities; or non-obstructive azoospermia; (2) recent use of medications known to significantly influence hormone profiles, including exogenous androgens and corticosteroids; (3) inability to provide qualified urine and semen samples. Detailed clinical information regarding reproductive system conditions—such as varicocele, cryptorchidism, history of genitourinary infections, testicular hydrocele, or sexually transmitted diseases—was collected for use as covariates or for sensitivity analyses. A total of 146 eligible men aged 22–57 years were included in the final analysis.

Sample collection procedures were standardized: urine and semen specimens were obtained during the same clinical visit using sterile polypropylene containers. Urine samples were immediately stored at –20 °C. Semen samples were collected via masturbation after 2 to 7 days of sexual abstinence. Following complete liquefaction at 37 °C within 60 minutes, aliquots of semen were frozen at –80 °C for subsequent laboratory analysis. In addition, blood samples were collected as part of routine clinical evaluation during assisted reproductive treatment, and serum reproductive hormone data were obtained from the participants’ electronic medical records. No additional blood samples were collected specifically for this study. In total, paired urine and semen samples were collected from 146 participants. Written informed consent was obtained from all individuals prior to inclusion. The study protocol was approved by the Ethics Committee of Shandong Maternal and Child Health Hospital (Approval No. 2024-116).

### 2.3 Hormones analysis

Serum reproductive hormone data, including total testosterone (total T), estradiol (E_2_), follicle-stimulating hormone (FSH), luteinizing hormone (LH), and prolactin (PRL), were retrieved from the participants’ electronic medical records. Blood samples were collected as part of routine clinical evaluation during assisted reproductive treatment. All hormone measurements were performed in the hospital’s clinical laboratory using an automated UniCel DxI 800 Access Immunoassay System (Beckman Coulter, Inc., Brea, CA, USA) according to standardized laboratory procedures. No additional blood collection or hormonal assays were performed specifically for this study. The total T/LH ratio, an established indicator of Leydig cell function, was subsequently calculated for statistical analysis.

### 2.4 Sample extraction and instrumental analysis

Urine and semen samples were processed according to a previously established protocol (29). Briefly, 1.0 mL of sample was placed into a polypropylene centrifuge tube, spiked with 10 ng internal standards and 82 units of β-glucuronidase, then incubated at 37 °C with shaking for over 12 hours. Following incubation, samples were extracted three times with 3.0 mL ethyl acetate each, involving vertexing, sonication, and centrifugation at 3000 ×g for 10 minutes. The combined supernatants (~9 mL) were evaporated under nitrogen and reconstituted in 1.0 mL methanol for analysis.

Target analytes were quantified using a Waters ACQUITY UPLC-TQS-MS/MS system equipped with electrospray ionization (ESI^+^). Chromatographic separation was performed on an ACQUITY UPLC BEH C18 column (100 mm × 2.1 mm, 1.7 µm) at room temperature. The mobile phases consisted of 0.1% formic acid in water (A) and acetonitrile (B). A gradient elution was applied as described in **Supplementary Table 2**, with a constant flow rate of 0.30 mL/min and an injection volume of 5 µL. The system dwell volume was approximately 300 µL, and the gradient was designed to account for this delay, ensuring stable baseline conditions for early-eluting analytes. Multiple reaction monitoring (MRM) in positive electrospray ionization mode was conducted. Detailed quantification parameters are provided in **Supplementary Table 3**, and representative chromatograms of NEOs and ISs are provided in **Supplementary Figure 1**.

### 2.5 Quality assurance and quality control

Ten procedural blanks were analyzed to correct for background contamination, and instrumental blanks were run every 20 samples to monitor carryover. Analyte identification was confirmed by retention time and ion transitions. Calibration curves (0–200 ng/mL) demonstrated excellent linearity (R² > 0.99) (**Supplementary Table 3**). To evaluate the quantitative performance of the validated method, the limit of detection (LOD) and limits of quantification (LOQ) were provided. The LOD and LOQ were based on the lowest concentration of the spiked levels with a signal-to-noise (S/N) ratio of 3:1 and 10:1 (30). Method detection limits (MDLs) and method quantification limits (MQLs) were set as 3 and 10 times, respectively, the standard deviation of target analyte levels in procedural blanks (31). The LODs for the analytes ranged from 0.003 to 0.089 ng/mL in urine and 0.021 to 1.087 ng/mL in semen. Accuracy and precision of the analytical method were evaluated through spiked recovery experiments. For urine, mean recoveries ranged from 87.25% DM-ACE to 110.00% (IMI-urea, IMIT), with relative standard deviations (RSDs) between 7.80% (CLO) and 21.65% (5-Hydroxy-imidacloprid, 5-OH-IMI). For semen, recoveries ranged from 75.99% (CLO) to 129.85% (5-OH-IMI), with RSDs from 5.02% (UF) to 12.79% (CLO). Values below LOD were treated as non-detectable (ND) and imputed by either (i) LOD/2 for compounds with detection frequency (DF) >50%, or (ii) f × LOD for compounds with DF <50%, where f is the proportion of detectable samples (32, 33). Urinary analyte concentrations were adjusted for dilution using specific gravity (SG) according to the formula: C_SG_=C×(SG_mean_)/(SG-1), where C_SG_ ​is the SG-adjusted concentration, C is the measured concentration, SG is the individual sample’s specific gravity, and SG_mean_ is the population mean specific gravity (34). SG-corrected concentrations were used for all subsequent analyses.

### 2.6 Exposure assessment in the populations

The estimated daily intake (EDI, ng/kg body weight/day) of urinary NEOs and their metabolites by young adults was calculated by Equation 1 and Equation 2 through previously reported method (35), as follows:

(1) $$EDI=\frac{C_{SG}\times V}{BW\times F_{ue}}\times 1000\;\;parent\;NEOs\;based$$

(2) $$EDI=\frac{C_{SG}\times V}{BW\times F_{ue}}\times \frac{M_{parent}}{M_{metabolism}}\times 1000\;\;metabolism\;based$$

where EDI (ng/kg bw/day) is the estimated daily intake of individual NEOs and their metabolites; *C* (ng/mL) is the urinary concentration of individual NEOs and their metabolites; *V* (L/day) is the volume of daily urine excretion, with 1.6 L/day for males (36), respectively; *BW* (kg) is the body weight; *F_ue_* the urinary excretion fraction of the respective NEOs and their metabolism (**Supplementary Table 4**), and and *M*_parent_ and *M*_metabolite_ = the molar masses of the parent NEOs and their metabolism (g/mol).

### 2.7 Covariate assessment

At enrollment, participants completed structured questionnaires collecting demographic data, lifestyle habits, and occupational status. Potential confounders were selected based on prior studies and directed acyclic graph (DAG) analysis (**Supplementary Figure 2**). The final covariates included age (years), body mass index (BMI, kg/m²), education level (below bachelor’s vs. bachelor’s or higher), smoking status (yes/no), season of sample collection (spring, summer, autumn), reproductive disease (yes/no) and occupational exposure risk (low vs. high).

### 2.8 Statistical analysis

Descriptive statistics were first conducted for participants’ demographic characteristics, urinary and seminal concentrations of NEOs and m-NEOs, and serum hormone levels. The distribution of continuous variables was evaluated and found to be non-normal. Accordingly, continuous variables were summarized using medians with interquartile ranges (P25, P75), whereas categorical variables were presented as frequencies and percentages. NEO concentrations were further described by their minimum, maximum, and quartiles. Outliers, defined as values below Q1 − 1.5×IQR or above Q3 + 1.5×IQR, were treated as missing values, and multiple imputation was applied for missing data. For the primary analyses, compounds with a DF >50% were modeled as continuous variables, whereas those with DF ≤50% were dichotomized (detected vs. undetected). This strategy was adopted to improve estimate stability and reduce exposure misclassification for chemicals with relatively low detection frequencies. Due to skewed distributions, NEO concentrations and hormone levels were natural log-transformed before correlation and regression analyses.

The Wilcoxon signed-rank test was applied to assess differences in NEO concentrations between paired urine and semen samples. Urine-to-semen concentration ratios were subsequently calculated to characterize the internal distribution and elimination patterns of each compound. To explore patterns of relative enrichment and co-distribution among compounds, hierarchical clustering was conducted based on the key distribution metrics (geometric mean, P25, median, P75, and P95). Hierarchical clustering using Ward’s method and Euclidean distance was applied, and dendrograms were visually inspected to identify groups of compounds with similar distribution profiles. Spearman’s rank correlation analysis was conducted to examine associations between neonicotinoids and their metabolites within urine, within semen, and across samples.

An Exposome-wide association study (EWAS) was performed to investigate associations between individual exposures and hormone outcomes. Urinary and seminal exposures were analyzed separately for each outcome using linear regression models adjusted for relevant covariates. For each exposure–outcome pair, effect estimates (β) was obtained, and percentage changes in outcomes per unit increase in exposure were calculated as (e^β − 1) × 100 with 95% confidence intervals (CIs). Multiple testing correction was performed using the Benjamini–Hochberg false discovery rate (FDR), with significance defined as an adjusted *P*-value <0.05.

The Deletion–Substitution–Addition (DSA) algorithm was applied to identify exposure biomarkers associated with each outcome. Starting from a base model containing pre-specified covariates, urinary and seminal exposures were entered as candidate predictors. The algorithm iteratively added, deleted, or substituted variables to minimize prediction error, with five-fold cross-validation used for model evaluation. A maximum of 15 predictors, one interaction term, and first-order polynomials were allowed. To ensure stability against random data partitioning, the procedure was repeated 1000 times with different random seeds, and variables selected in at least 5% of iterations were considered robust. These exposures, together with forced-in covariates, were subsequently included in the final multivariable linear regression models, with results expressed as mean differences and 95% CIs.

For mixture assessment, DF >70% was used as a predefined inclusion criterion. Eligible urinary compounds were natural log-transformed, standardized (z-scores), and jointly modeled using quantile g-computation (QGC) with quartile categorization (q=4). This approach estimates both the overall mixture effect and the relative contribution of each component, while accounting for correlations among exposures and potential confounding influences (37).

### 2.9 Sensitive analysis

Several sensitivity analyses were conducted to assess the robustness of the findings. First, multivariable linear regression models were fitted as an alternative approach to evaluate the associations between exposures and hormone outcomes. Second, for both EWAS and DSA analyses, participants with diagnosed reproductive disorders (n=107) were excluded to minimize potential bias from underlying clinical conditions. Third, additional EWAS and DSA analyses were performed after excluding compounds with DFs <30% to evaluate the influence of infrequently detected chemicals on the study findings. Finally, weighted quantile sum (WQS) regression was applied as an additional mixture modeling strategy to complement the QGC analysis.

## 3 Result

### 3.1 Demographic characteristics and hormones level of the participants

The baseline characteristics of the participants (N = 146) are summarized in **Table 1**. Median age was 34.0 years and BMI 26.1 kg/m². Samples were mostly collected in spring (46.6%) and summer (43.8%). Occupational exposure was low in 57.5% and high in 42.5%, and 64.4% had a bachelor’s degree or higher. A total of 73.3% reported no reproductive disorders, and 74.7% were non-smokers. Median hormone levels were FSH 4.09 IU/L, LH 3.81 IU/L, T 11.2 nmol/L, PRL 18 ng/mL, E_2_ 52 pg/mL, and T/LH ratio 2.58.

**Table 1:** Demographic characteristics and reproductive hormones level of the study population.

Characteristic	N = 146
Age, years, Median (Q1, Q3)	34.0 (31.0, 38.0)
BMI, kg/m^2^, Median (Q1, Q3)	26.1 (23.9, 28.4)
Season, n (%)	
Autumn	14 (9.59%)
Spring	68 (46.58%)
Summer	64 (43.84%)
Occupation, n (%)	
High	62 (42.47%)
Low	84 (57.53%)
Education, n (%)	
Bachelor’s degree or above	94 (64.38%)
Below bachelor’s degree	52 (35.62%)
Reproductive disease, n (%)	
No	107 (73.29%)
Yes	39 (26.71%)
Smoke, n (%)	
No	109 (74.66%)
Yes	37 (25.34%)
FSH, IU/L, Median (Q1, Q3)	4.09 (3.08, 5.13)
LH, IU/L, Median (Q1, Q3)	3.81 (2.99, 5.05)
T, nmol/L, Median (Q1, Q3)	11.2 (4.4, 15.4)
PRL, ng/mL, Median (Q1, Q3)	18 (10, 181)
E_2_, pg/mL, Median (Q1, Q3)	52 (33, 117)
T/LH, Median (Q1, Q3)	2.58 (1.26, 3.86)

BMI, body mass index; FSH, follicle-stimulating hormone; E_2_, estradiol; PRL, prolactin; T, testosterone; LH, luteinizing hormone; T/LH, testosterone-to-LH ratio;.

### 3.2 Urine and semen concentrations of neonicotinoids and their metabolites

**Table 2** presents the concentration distribution of NEOs and m-NEOs in urine and semen. In urine, detection frequencies ranged from 43.15% (DM-THM) to 100% (ACE). DN (median 1.03 ng/mL) and DM-ACE (median 0.58 ng/mL) were predominant. In semen, detection varied from 19.86% (IMI) to 99.32% (DN). Several compounds (UF, DN-IMI-olefin, 5-OH-IMI, DNT, IMI, IMIT) were present in fewer than half of samples, while DN showed the highest median concentration (3.26 ng/mL).

**Table 2:** Descriptive statistics of neonicotinoid and their metabolite concentrations detected in urine and semen (ng/mL).

Chemicals (ng/ml)	Urine					Semen				
	DF (%)	Median	P25	P75	Range	DF (%)	Median	P25	P75	Range
ACE	100.0	0.08	0.06	0.12	0.03 ~ 0.80	93.15	0.54	0.19	1.12	ND ~ 17.88
CLO	99.32	0.48	0.25	0.84	ND ~ 186.84	66.44	0.24	ND	0.40	ND ~ 2.12
DNT	–	–	–	–	–	45.21	ND	ND	1.82	ND ~ 9.07
IMI	93.84	0.14	0.09	0.29	ND ~ 3.59	19.86	ND	ND	ND	ND ~ 3.05
IMIT	92.47	0.09	0.06	0.14	ND ~ 7.83	43.15	ND	ND	0.28	ND ~ 1.06
THCP	59.59	0.02	ND	0.04	ND ~ 0.72	–	–	–	–	–
DN-IMI-olefin	93.15	0.11	0.07	0.19	ND ~ 5.17	46.58	ND	ND	0.09	ND ~ 0.26
DM-ACE	94.52	0.58	0.38	2.02	ND ~ 142.70	59.59	0.13	ND	0.24	ND ~ 3.45
DN-IMI	97.95	0.25	0.06	3.25	ND ~ 39.30	89.04	0.11	0.04	0.21	ND ~ 1.26
IMI-urea	85.62	0.10	0.06	0.14	ND ~ 26.38	85.62	0.97	0.54	1.44	ND ~ 15.16
5-OH-IMI	81.51	0.20	0.11	0.34	ND ~ 41.88	27.40	ND	ND	0.92	ND ~ 5.36
DM-THM	43.15	ND	ND	0.52	ND ~ 4.83	–	–	–	–	–
DN	99.32	1.03	0.79	1.36	ND ~ 8.03	99.32	3.26	2.64	4.20	ND ~ 7.83
UF	84.25	0.27	0.13	0.52	ND ~ 12.24	43.84	ND	ND	0.42	ND ~ 1.01

DN, Desnitro-dinotefuran; UF, Dinotefuran-urea; DM-ACE, Desmethyl-acetamiprid; DN-IMI-olefin, Desnitro-imidacloprid-olefin; DN-IMI, Desnitro-imidacloprid; IMI-urea, Imidacloprid-urea; 5-OH-IMI, 5-Hydroxy-imidacloprid; DM-THM, Desmethyl-thiamethoxam; DNT, Dinotefuran; ACE, Acetamiprid; CLO, Clothianidin; THCP, Thiacloprid; IMI, Imidacloprid; IMIT, Imidaclothiz; DF, detection frequency. Note: Among the 18 target analytes, 14 were successfully quantified in at least one biological matrix (urine or semen). Four compounds (FLO, THM, Descynao-ACE, and THCP-amide) were excluded due to analytical limitations and are not presented here.

**Figure 1:**
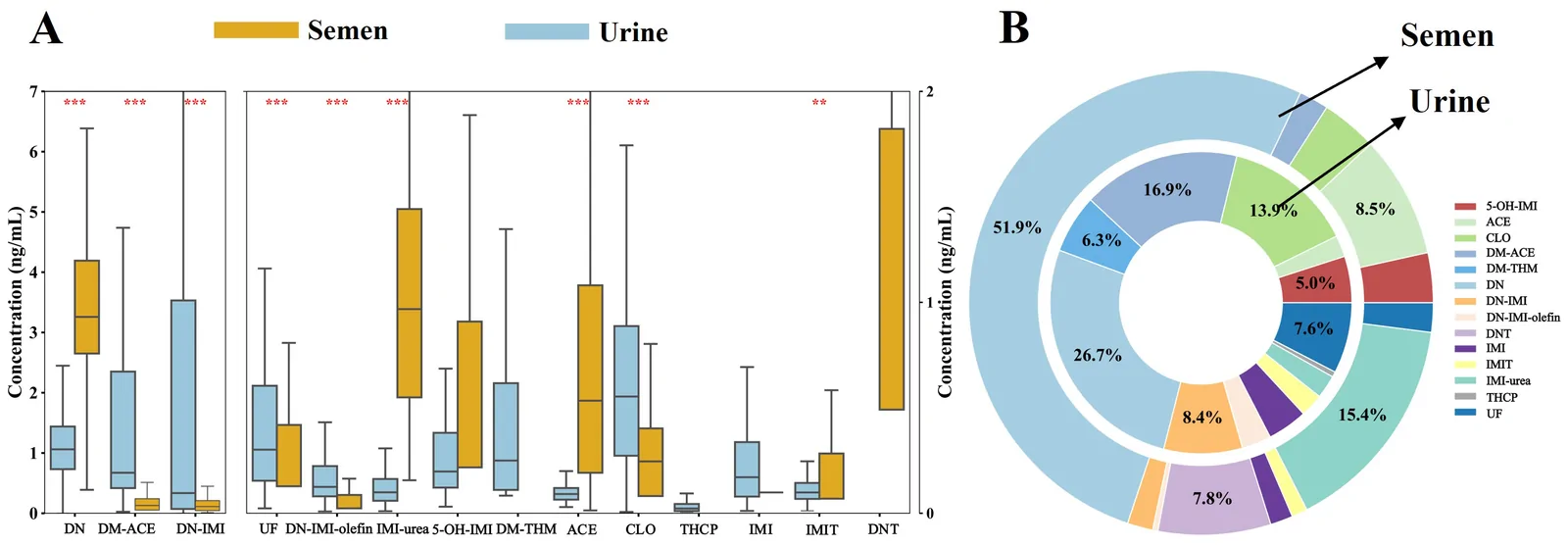
Comparison of concentrations of Neonicotinoids (NEOs) and their metabolites (m-NEOs) in paired urine and semen samples **(A)** and composition ratios of Neonicotinoids and their metabolites in urine and semen **(B)**. **P < 0.01, ***P < 0.001.

Comparisons between matrices (**Figure 1A**) showed that DM-ACE, UF, DN-IMI-olefin, CLO, and DN-IMI were significantly higher in urine, whereas DN, ACE, IMI-urea, and IMIT were significantly higher in semen (all *P* < 0.05). Compositionally, urine was dominated by DN (26.7%), DM-ACE (16.9%), and CLO (13.9%), while semen was mainly DN (51.9%) and ACE (15.4%) (**Figure 1B**).

### 3.3 Partitioning characteristics of neonicotinoids and their metabolites between urine and semen

**Figure 2:**
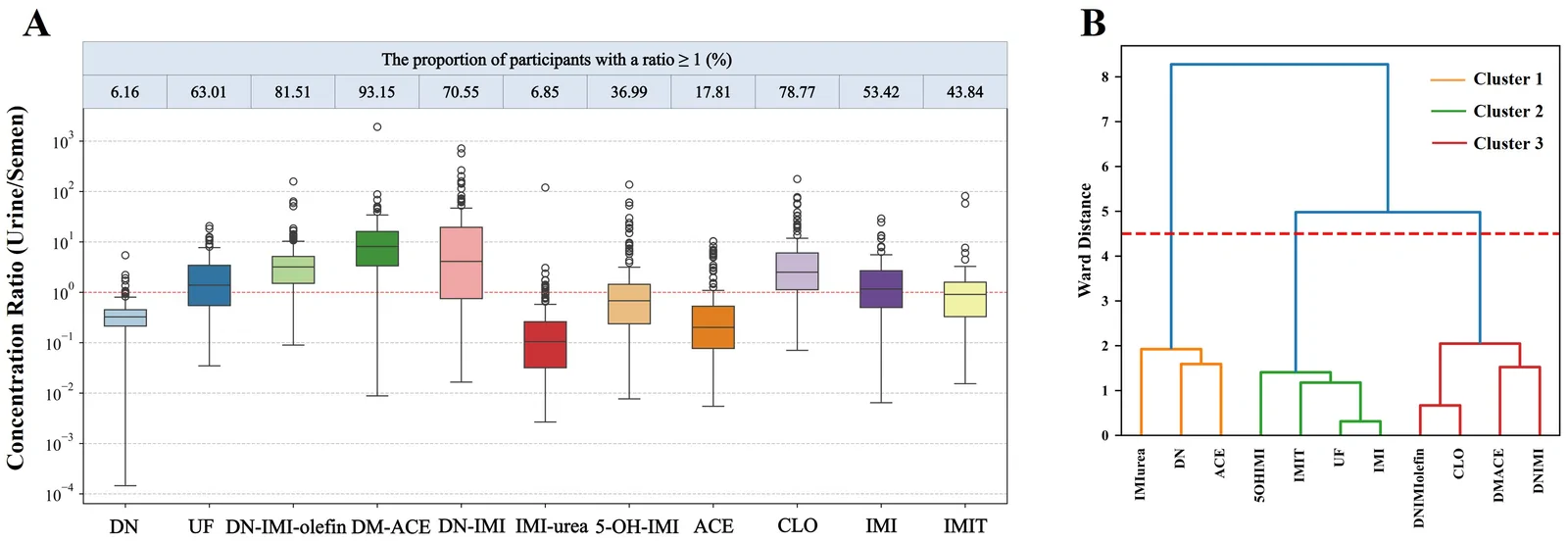
Concentration ratios of Neonicotinoids and their metabolites in urine and semen. **(A)** Urine-to-semen concentration ratios of each compound. **(B)** Hierarchical clustering of compounds based on their concentration ratios.

**Figure 3:**
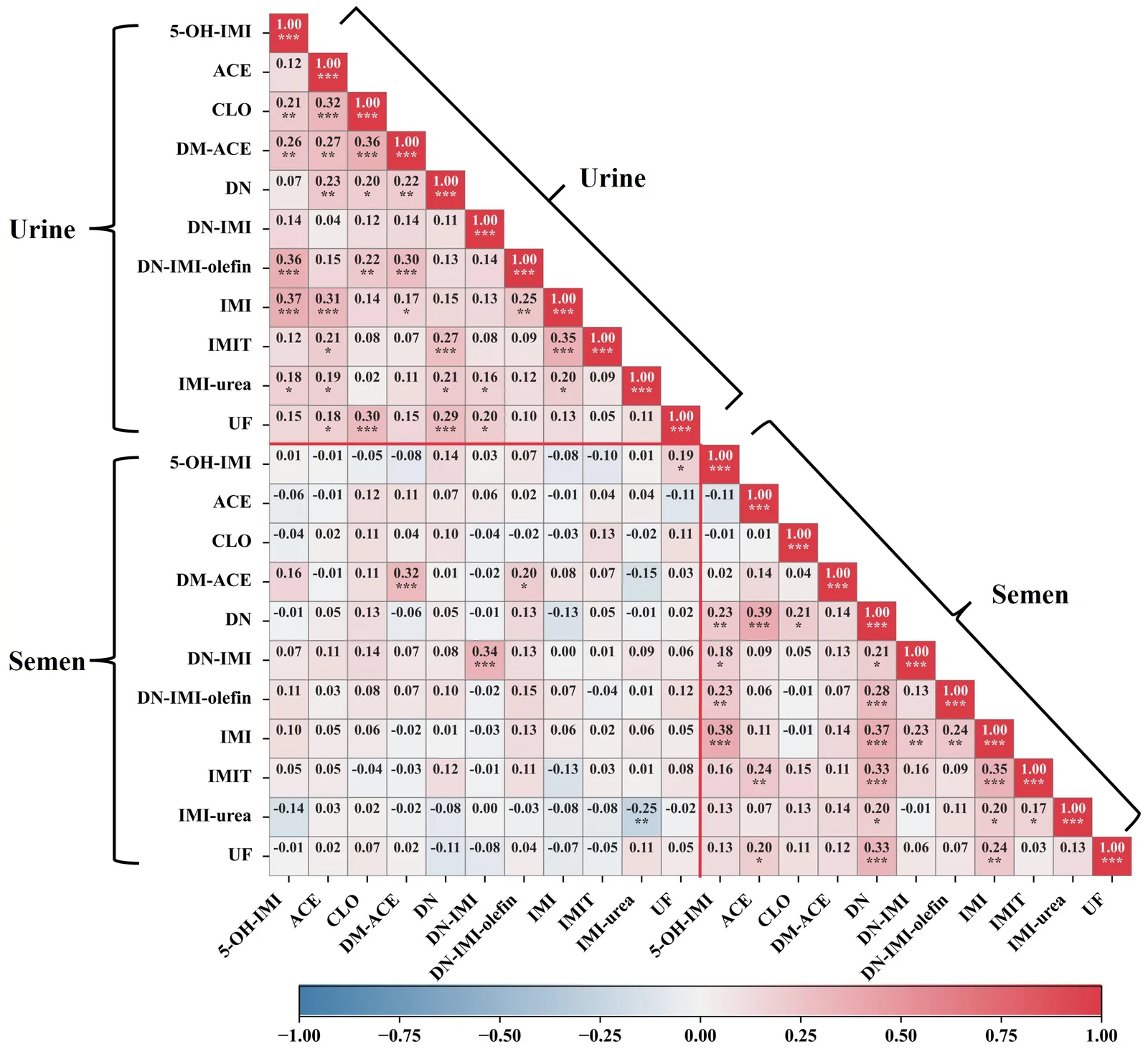
Correlation heat map of Neonicotinoids and their metabolites in urine and semen. *P < 0.05, **P < 0.01, ***P < 0.001.

The urine-to-semen concentration ratios of NEOs and m-NEOs were calculated to assess their distribution characterizes between the two matrices (**Supplementary Table 5**). As shown in **Figure 2A**, 6.85 to 93.15% of the participants had urine-to-semen concentration ratios higher than 1 for each compound. **Figure 2B** shows hierarchical clustering of NEOs and m-NEOs based on urine-to-semen concentration ratios, identifying three distinct clusters: Cluster 1 (DN, ACE, IMI-urea) with low ratios (<0.33) indicates relative enrichment in semen. Cluster 2 (UF, IMI, IMIT, 5-OH-IMI) with intermediate ratios (~0.7–1.4) suggests similar distribution in urine and semen. Cluster 3 (DN-IMI-olefin, DN-IMI, DM-ACE, CLO) with high ratios (>2.5) reflects predominant urinary excretion. These clusters highlight differences in excretion patterns and tissue distribution among compounds. Correlation analyses (**Figure 3**) showed weak-to-moderate positive associations within urine (r = 0.09–0.37), weaker within semen (r = –0.01–0.39), and limited cross-matrix correlations.

### 3.4 Risk and exposure assessment

Estimated daily intakes (EDIs) were derived for ACE, DM-ACE, IMI, 5-OH-IMI, and CLO (**Supplementary Table 6**). Among parent compounds, ACE exhibited the highest median EDI (67.90 ng/kg bw/day), followed by IMI (28.28 ng/kg bw/day) and CLO (16.41 ng/kg bw/day). For metabolites, DM-ACE showed the highest median EDI (50.65 ng/kg bw/day), whereas 5-OH-IMI was lower (12.33 ng/kg bw/day). When compared with their corresponding acceptable daily intake (ADI) values (**Supplementary Table 4**), the median and P95 EDI estimates remained several orders of magnitude lower for all quantified compounds (e.g., ACE P95 = 287.24 vs. ADI = 5000 ng/kg bw/day; IMI P95 = 123.68 vs. ADI = 60000 ng/kg bw/day; CLO P95 = 280.47 vs. ADI = 97000 ng/kg bw/day).

### 3.5 Association of NEOs and metabolites with reproductive hormones level

**Figure 4:**
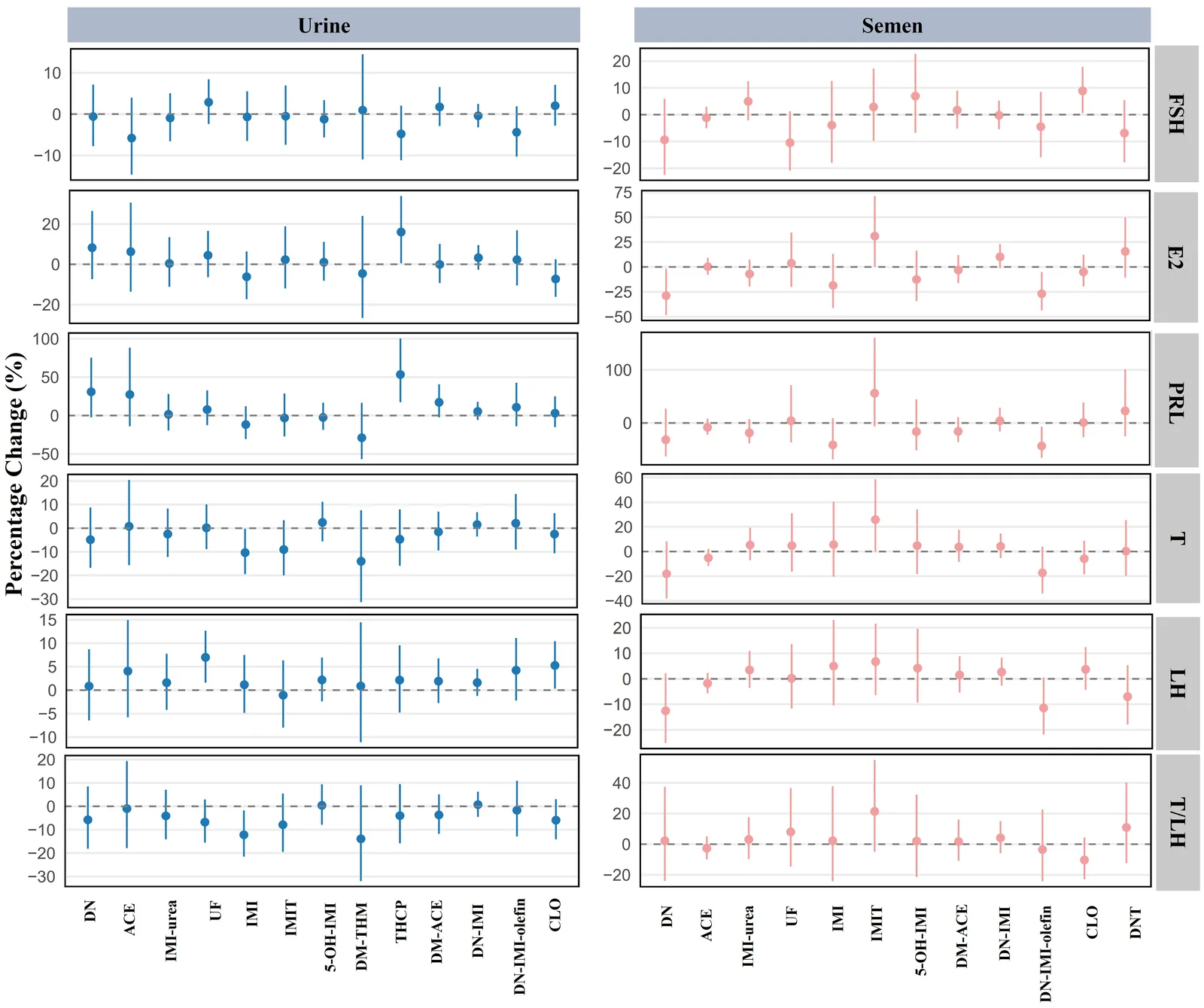
Associations between urinary/seminal neonicotinoid concentrations and male reproductive hormone levels using EWAS models. The models were adjusted for age, BMI, season, smoking status, reproductive disease, occupation and education.

In the EWAS analysis, several nominal associations were observed between NEOs and reproductive hormone levels (**Figure 4**; **Supplementary Table 7**). In semen, each unit increase in CLO concentration was associated with an 8.88% (95% CI: 0.59%–17.85%, *P* = 0.037) higher FSH level. Conversely, the detection of DN-IMI-olefin was linked to a 43.33% reduction in PRL (95% CI: –65.55% to –6.77%, *P* = 0.027), while DN and DN-IMI-olefin were also inversely associated with E_2_, showing 28.82% (95% CI: –48.40% to –1.82%, *P* = 0.040) and 26.90% (95% CI: –43.67% to –5.13%, *P* = 0.020) lower levels, respectively. In urine, UF and CLO concentrations were associated with 6.98% (95% CI: 1.59%–12.66%, *P* = 0.012) and 5.26% (95% CI: 0.33%–10.43%, *P* = 0.038) higher LH levels, respectively, whereas IMI concentration was associated with a 10.36% (95% CI: -19.47%–0.21%, *P* = 0.048) lower T level and a 12.18% reduction in the T/LH ratio (95% CI: –21.50% to –1.76%, *P* = 0.025). Additionally, THCP exposure was positively associated with PRL (53.37% increase, 95% CI: 17.40%–100.36%, *P* = 0.002) and E_2_ (16.00% increase, 95% CI: 0.52%–33.86%, *P* = 0.044). However, none of these associations remained statistically significant after correction for multiple testing using the FDR method.

In the DSA analysis, several NEO exposures were repeatedly selected as predictors for reproductive hormone levels, with selection frequencies ranging from 12% to 92% (**Table 3**). Among semen biomarkers, CLO was positively associated with FSH (β = 0.09, 95% CI: 0.01–0.17), whereas UF was negatively associated with FSH (β = -0.13, 95% CI: -0.25–0.01). For E_2_, multiple compounds showed differential associations: DN exhibited a negative association (β = -0.65, 95% CI: -1.00–0.31), while IMIT (β = 0.38, 95% CI: 0.11–0.65), DN-IMI (β = 0.16, 95% CI: 0.05–0.27), and DNT (β = 0.28, 95% CI: 0.03–0.53) were positively associated. IMI showed a nonsignificant negative trend with E_2_ (β = -0.30, 95% CI: -0.64–0.06). Among urinary exposures, THCP was positively associated with PRL (β = 0.43, 95% CI: 0.16–0.70) and E_2_ (β = 0.16, 95%CI: 0.03–0.30), UF with LH (β = 0.07, 95% CI: 0.02–0.12), and IMI was negatively associated with the T/LH ratio (β = -0.13, 95% CI: -0.24–0.02). These results indicate that specific neonicotinoid compounds may differentially influence reproductive hormones depending on the biomarker matrix (semen versus urine).

**Table 3:** Associations between neonicotinoid exposures and reproductive hormones in DSA analysis.

Chemical	Sample	DSA frequency (%)	FSH		E_2_		PRL		T		LH		T/LH	
			β	95% CI	β	95% CI	β	95% CI	β	95% CI	β	95% CI	β	95% CI
CLO	Semen	27.9	**0.09**	**0.01, 0.17**	–	–	–	–	–	–	–	–	–	–
UF	Semen	17.9	**-0.13**	**-0.25, -0.01**	–	–	–	–	–	–	–	–	–	–
DN	Semen	20.6	–	–	**-0.65**	**-1.00, -0.31**	–	–	–	–	–	–	–	–
IMIT	Semen	20.6	–	–	**0.38**	**0.11, 0.65**	–	–	–	–	–	–	–	–
DN-IMI	Semen	19.2	–	–	**0.16**	**0.05, 0.27**	–	–	–	–	–	–	–	–
DNT	Semen	16.3	–	–	**0.28**	**0.03, 0.53**	–	–	–	–	–	–	–	–
IMI	Semen	12.3	–	–	-0.30	-0.64, 0.04	–	–	–	–	–	–	–	–
THCP	Urine	23.6/92.3	–	–	**0.16**	**0.03, 0.30**	**0.43**	**0.16, 0.70**	–	–	–	–	–	–
UF	Urine	12.3	–	–	–	–	–	–	–	–	**0.07**	**0.02, 0.12**	–	–
IMI	Urine	35.7	–	–	–	–	–	–	–	–	–	–	**-0.13**	**-0.24, -0.02**

FSH, follicle-stimulating hormone; E_2_, estradiol; PRL, prolactin; T, testosterone; LH, luteinizing hormone; T/LH, testosterone-to-LH ratio; DN, Desnitro-dinotefuran; UF, Dinotefuran-urea; DN-IMI, Desnitro-imidacloprid; CLO, Clothianidin; THCP, Thiacloprid, IMI, Imidacloprid, IMIT, Imidaclothiz. Bold = significant at the 5% level. β (95% CI) estimated from final DSA models adjusted for age, BMI, season, smoking status, reproductive disease, occupation and education. DSA frequency = proportion of times the chemical was selected across 1000 resampling iterations. “–” indicates the chemical was not selected for the outcome in the final model.

Comparison of DSA and EWAS results revealed that exposures most frequently selected by DSA generally corresponded to nominally significant associations in EWAS. Notably, semen CLO–FSH, DN–E_2_, and urine UF–LH, THCP–PRL were consistently highlighted across both approaches, suggesting robust exposure–hormone relationships despite no associations reaching significance after multiple-testing correction.

**Figure 5:**
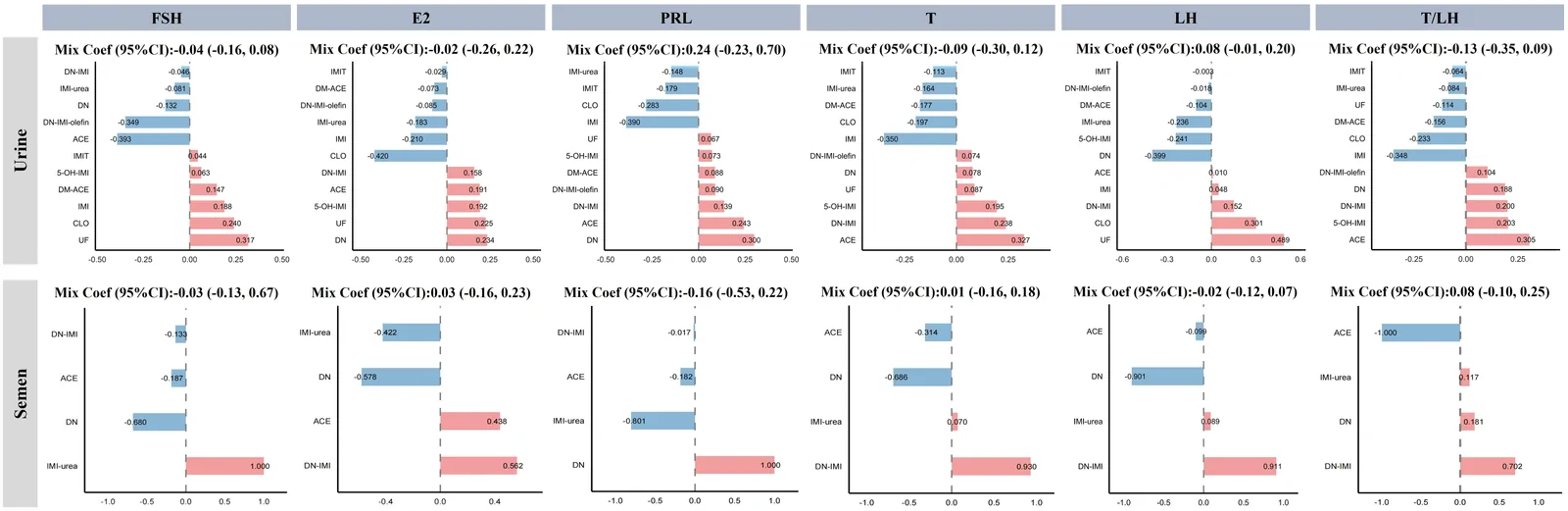
Quantile g-computation analysis of the effects of urinary and seminal Neonicotinoids and their metabolites on reproductive hormones level. The models were adjusted for age, BMI, season, smoking status, reproductive disease, occupation and education.

We further evaluated the associations of the joint exposure to NEOs and m-NEOs in urine and semen with reproductive hormones level using the QGC models (**Figure 5**). QGC model analyses indicated that urine exposure was not significantly associated with male reproductive hormone levels. Specifically, LH (β = 0.08, 95% CI: -0.01, 0.20) and PRL (β = 0.24, 95% CI: -0.23, 0.70) exhibited slight positive trends, whereas FSH (β = -0.04, 95% CI: -0.16, 0.08), T (β = -0.09, 95% CI: -0.30, 0.12), E_2_ (β = -0.02, 95% CI: -0.26, 0.22), and T/LH (β = -0.13, 95% CI: -0.35, 0.09) showed slight negative trends, none of which reached statistical significance. Similarly, semen exposure did not demonstrate significant effects. Slight positive trends were observed for E_2_ (β = 0.03, 95% CI: -0.16, 0.23), T (β = 0.01, 95% CI: -0.16, 0.18), and T/LH (β = 0.08, 95% CI: -0.10, 0.25), whereas FSH (β = -0.03, 95% CI: -0.13, 0.67), PRL (β = -0.16, 95% CI: -0.53, 0.22), and LH (β = -0.02, 95% CI: -0.12, 0.07) exhibited slight negative trends.

### 3.6 Sensitivity analysis

Sensitivity analyses largely confirmed the primary findings. In multivariable linear regression (**Supplementary Table 8**; **Supplementary Figure 3**), seminal CLO was positively associated with FSH (β = 0.09, 95% CI: 0.01–0.16), while UF showed a negative trend (β = −0.11, 95% CI: −0.24–0.01). DN was inversely associated with E_2_, whereas IMIT and DN-IMI showed positive trends. In urine, THCP was positively associated with PRL (β = 0.43, 95% CI: 0.16–0.70), and UF and CLO with LH. None remained significant after FDR correction, but results supported robustness of the main findings.

Excluding participants with reproductive disorders (N = 107) did not materially change EWAS results, and no associations reached FDR significance (**Supplementary Table 9**). DSA findings were also largely confirmed (**Supplementary Table 10**): CLO in semen remained positively associated with FSH (β = 0.10, 95% CI: 0.01–0.20), seminal IMIT with estradiol (β = 0.42, 95% CI: 0.11–0.72), UF in urine with LH (β = 0.08, 95% CI: 0.02–0.14), and THCP in urine with PRL (β = 0.59, 95% CI: 0.29–0.88) and nominally with E_2_ (β = 0.16, 95% CI: −0.01–0.32). Additional associations included seminal IMI with PRL (β = −1.04, 95% CI: −1.78–−0.29), seminal IMIT with PRL (β = 0.91, 95% CI: 0.33–1.49), seminal IMIT with T (β = 0.29, 95% CI: 0.03–0.55), and urinary DN-IMI-olefin with LH (β = 0.08, 95% CI: 0.01–0.15). The previously strong DN–E_2_ association was no longer observed.

Further sensitivity analyses excluding compounds with DF <30% did not materially change the observed EWAS associations. Similarly, the DSA analysis yielded comparable results, with the primary exposure biomarkers remaining consistently identified (**Supplementary Table 11**).

WQS regression for urine and semen (**Supplementary Table 12**) yielded results consistent with QGC. Most hormone estimates were near null, with only urinary LH showing a nominally significant negative association (β = 0.51, 95% CI: 0.14–0.88). ACE, DN-IMI, IMI-urea, and CLO were major contributors, varying by hormone and direction. These findings support the robustness of the main analysis.

## 4 Discussion

In this study, we characterized NEOs and m-NEOs in paired urine and semen samples and observed distinct partitioning profiles across matrices. Detection frequencies and concentrations were generally higher in urine, consistent with its role as the primary excretion pathway (1, 38). DN—a metabolite of DNT—was the most abundant compound in both matrices (urine: 1.03 ng/mL; semen: 3.26 ng/mL), followed by DN-IMI-olefin in urine (0.58 ng/mL). When compared with earlier biomonitoring data, our observed levels appear elevated. Previous population-based studies reported urinary medians of IMI at 0.21 ng/mL, CLO at 0.24 ng/mL, and DIN at 0.14 ng/mL (39), substantially lower than the DN and metabolite concentrations in our cohort. Another study measuring multiple metabolites, including 5-OH-IMI and Of-IMI, reported urinary medians of 0.42–1.02 ng/mL (40), which again fall below the DN and DM-ACE levels identified here. These differences may reflect higher exposure in our study population or variation in metabolism and clearance pathways. The striking predominance of DN in semen, despite limited toxicological evaluation, raises concern for selective retention or bioaccumulation within reproductive tissues and underscores the need for further investigation.

Importantly, the predominance of metabolites over parent compounds in urine is fully consistent with our findings (for example, IMI-related metabolites and DM-ACE meeting or exceeding their respective parents at the median) and aligns with growing biomonitoring evidence. Controlled human dosing and excretion studies demonstrate that for IMI the primary urinary biomarkers are not the parent compound but the Phase I metabolites—namely DN-IMI, imidacloprid-olefin (IMI-olefin), 5-OH-IMI, and IMI-urea. For instance, a controlled human metabolism study found extensive conversion of IMI to these metabolites, with signal intensities of metabolites substantially higher than parent in many cases (28). In a large Chinese cohort, the urinary concentrations of DN-IMI and IMI-olefin were 4–40 times higher than IMI itself, and together accounted for approximately 92% of the sum of IMI plus relevant metabolites (Σ 3IMI) in urine (41). Similarly, for ACE, human biomonitoring shows that the N-desmethyl metabolite DM-ACE frequently yields higher detection frequencies and concentrations than ACE itself (42). Collectively, these data justify shifting the focus of exposure assessment toward multi-metabolite panels (e.g., DN-IMI, IMI-olefin, 5-OH-IMI, IMI-urea, DM-ACE) instead of parent-only metrics, because urinary burdens of neonicotinoids (NEOs) reflect not only external exposure but also internal biotransformation and elimination kinetics. Moreover, recent literature emphasizes that relic reliance on parent compounds may seriously underestimate actual human exposure to NEOs (43).

The partitioning profiles observed further underscore these differences: DN, ACE, and IMI-urea showed relatively low urine-to-semen ratios, suggesting preferential distribution to, or retention within, the male reproductive tract, whereas DN-IMI-olefin, DM-ACE, and CLO were more efficiently excreted in urine. Such distribution patterns likely reflect distinct pharmacokinetic properties shaped by metabolism, polarity, lipophilicity, and protein-binding affinity. Specifically, Cluster 1 compounds may exhibit greater tissue retention due to relatively stronger interactions with reproductive tissues or seminal proteins, whereas Cluster 3 compounds, particularly oxidative metabolites, are generally more polar and therefore more readily eliminated through renal excretion. Cluster 2 compounds showed comparable concentrations in urine and semen, suggesting an intermediate partitioning behavior resulting from a balance between tissue distribution and urinary clearance. Although lipophilicity (e.g., log P) may contribute to membrane permeability and tissue partitioning, the observed clustering is likely driven by the combined effects of physicochemical properties and biotransformation rather than by any single parameter. Similar trends have been reported in urine–serum comparisons, where most NEOs exhibited urine-to-serum ratios >1.0, and metabolites such as 5-OH-IMI and Of-IMI reached levels several orders of magnitude higher than their parent IMI, consistent with preferential renal clearance (1). Paired blood–urine biomonitoring in China likewise showed lower blood-to-urine ratios for metabolites compared with parent compounds (42). The detection of NEOs in seminal plasma at concentrations distinct from urine (20, 25), combined with experimental evidence of high protein-binding affinity (44), supports the role of local secretion and selective retention in reproductive fluids. However, the biological mechanisms underlying the enrichment of DN, ACE, and IMI in semen remain unclear. Although the blood–testis barrier (BTB) is a dynamic and selectively permeable structure rather than an absolute barrier, direct evidence demonstrating that these specific NEOs can cross the BTB and enter the seminiferous tubule lumen is currently lacking (45, 46). Therefore, further mechanistic studies integrating paired blood, testicular tissue, and semen samples are warranted to elucidate the transport pathways responsible for NEO accumulation in the male reproductive tract. Moreover, weak intra-matrix correlations and limited cross-matrix concordance indicate that urinary and seminal concentrations capture distinct exposure and distribution dynamics. Together, these findings emphasize that multi-matrix sampling is crucial for accurate internal exposure assessment, particularly for compounds with potential reproductive toxicity.

In interpreting these results, it is important to consider whether each analyte represents a human metabolite or an exogenous compound. Based on recent work (26–28), IMI-urea, DN-IMI, and 5-OH-IMI are recognized as human metabolic products, whereas DN-IMI-olefin and descyano-ACE are more likely formed through abiotic transformation or microbial degradation before exposure. Consequently, elevated levels of these latter compounds in urine or semen reflect direct environmental exposure rather than enhanced human metabolic activity. This distinction is crucial, as the toxicokinetic and endocrine implications of exogenously derived species may differ from those of metabolites produced within the human body. Accordingly, we interpreted our findings for such compounds with greater caution and avoided over-attributing them to endogenous metabolism.

Beyond exposure characterization, both EWAS and DSA consistently identified nominal associations between specific NEOs and reproductive hormone alterations, although none survived multiple-testing correction. Nonetheless, the consistency across methods suggests biologically relevant signals. CLO and UF were positively associated with FSH and LH, aligning with prior reports of altered gonadotropins in pesticide-exposed populations (47, 48). Reduced testosterone associated with IMI exposure mirrors animal models (49, 50) and human data showing impaired spermatogenesis and testosterone suppression (16). Experimental studies support these findings: IMI reduced serum testosterone and increased FSH in male rodents, though LH remained unchanged (51). ACE may impair steroidogenesis through oxidative stress, promoting lipid peroxidation and reducing cholesterol availability for testosterone synthesis (52). CLO has been shown to interfere with estrogen biosynthesis and gonadotropin-releasing hormone signaling in female rodents (53), suggesting potential impacts on male endocrine function. The inverse associations of DN with E_2_ and of DM-ACE with PRL observed here also point toward endocrine disruption; *in vitro* studies report that DN-IMI can alter estrogen synthesis and secretion (54), although epidemiologic evidence remains absent. In addition, THCP exposure was positively associated with PRL and E_2_ concentrations in our study. To our knowledge, no previous epidemiological or experimental studies have specifically reported an association between THCP exposure and increased PRL or E_2_ levels. Therefore, these findings should be interpreted cautiously, and the underlying biological mechanisms require further investigation in independent populations and experimental models. These converging signals underscore the need for replication in larger cohorts.

Notably, our EWAS analysis revealed more nominal associations between IMI and male reproductive hormones in urine compared to semen, where no significant associations were observed. These matrix-specific disparities suggest that urinary and seminal concentrations likely capture distinct toxicokinetic dynamics, exposure windows, and biological characteristics. Specifically, urinary burdens reflect systemic elimination, while seminal concentrations may involve local secretion or selective retention. However, given that these nominal associations did not remain statistically significant after FDR correction, these findings should be interpreted with caution and require further validation in larger, longitudinal studies.

It should also be noted that several analytes measured in this study—particularly DN-IMI-olefin and DM-ACE—may not primarily arise from human metabolism. In such cases, their presence in biological fluids more likely represents direct exposure to the compound itself through diet or environmental sources. The concentration detected thus reflects external exposure magnitude rather than metabolic activity. For risk interpretation, these compounds serve better as exposure biomarkers, while human-formed metabolites (e.g., DN-IMI, IMI-urea) are more informative for assessing internal biotransformation and tissue distribution.

Several mechanisms could plausibly underlie these hormone alterations. Low-dose DN-IMI induces nAChR desensitization in neuronal cells, disturbing cholinergic signaling (55). NEOs and their metabolites also impair antioxidant defenses (e.g., superoxide dismutase, catalase), elevate reactive oxygen species, and cause oxidative stress (56, 57), damaging Leydig cells and impairing androgen production (54). Animal and *in vitro* studies further suggest NEOs accumulate in gonadal tissue, disrupt steroidogenic gene expression (Cyp17a1, Cyp19a1, Hsd3b1), and inhibit cytochrome P450 activity, thereby altering testosterone–estradiol balance (54, 58, 59). Desnitro metabolites such as DN-IMI can cross the blood–brain barrier and suppress hypothalamic GnRH release (60), disturbing hypothalamic–pituitary–gonadal signaling. Together, these mechanisms lend strong biological plausibility to the endocrine-disrupting potential of NEOs.

EDI values derived from urinary concentrations ranged from 44.02 ng/kg·day (IMI) to 190.90 ng/kg·day (DM-ACE). These screening-level estimates are several orders of magnitude lower than the acceptable daily intakes reported by EFSA and JECFA (e.g., CLO 97000 ng/kg·day; IMI 60000 ng/kg·day; ACE 5000 ng/kg·day), and are therefore used solely to contextualize population exposure levels rather than to perform a quantitative risk assessment. Due to the lack of reliable, compound-specific urinary excretion fractions for neonicotinoids in humans, EDIs were not calculated for all parent compounds. Nonetheless, this limitation does not affect the validity of relative comparisons across compounds or individuals, nor does it hinder the interpretation of exposure patterns. Consistent EDI ranges have been reported in previous biomonitoring studies (e.g., ~0.5–52.8 ng/kg bw/day in young adults in China (61)), supporting the use of this approach as a screening-level exposure indicator.

The strengths of this study lie in its novelty and methodological rigor. To our knowledge, it is the first to simultaneously measure and compare a broad spectrum of NEOs and m-NEOs in both urine and semen. This dual-matrix design reduces exposure misclassification and provides more accurate insight into reproductive risk. Secondly, the application of multiple advanced statistical approaches (EWAS, DSA, QGC, and WQS) enabled a robust and comprehensive evaluation of exposure–hormone associations, strengthening the credibility of the nominally significant findings.

Despite these strengths, several limitations should be acknowledged. The cross-sectional design precludes causal inference, and single-time-point measurements may not fully capture temporal variability in exposures or hormone levels. Although we adjusted for key demographic and lifestyle factors, the absence of dietary information and other unmeasured confounders—such as participants’ lifestyle and agricultural or industrial exposure—could influence hormone concentrations. In particular, dietary information was not collected in the present study, limiting our ability to evaluate the contribution of dietary sources to NEOs and m-NEOs exposure and their potential effects on reproductive hormones. Future studies should include these variables to better assess their potential contribution and to improve the robustness of the findings. In addition, NEOs and m-NEOs concentrations were not measured in blood or serum in the present study. Although urine and semen were selected as matrices to characterize environmental exposure and potential reproductive effects, the absence of blood-based measurements limited our ability to evaluate systemic exposure levels and directly examine the relationships between circulating NEOs/m-NEOs concentrations and serum reproductive hormones. Future studies incorporating paired blood, urine, and semen samples are warranted to better elucidate the toxicokinetic profiles and endocrine-disrupting mechanisms of these compounds. Furthermore, potential selection bias due to the recruitment of men seeking ART services may limit the generalizability of our findings to the broader male population. Future studies involving population-based cohorts are warranted to validate these associations. Finally, while several nominal associations were observed, none remained statistically significant after multiple-testing correction, underscoring the need for larger, longitudinal studies with repeated biomarker measurements to confirm and clarify these relationships.

## 5 Conclusion

In conclusion, NEOs and m-NEOs are widely detectable in urine and semen, with compound-specific distribution patterns. DN and its metabolites were notably enriched in semen, suggesting selective retention in reproductive tissues. While no associations with reproductive hormones remained significant after multiple-testing correction, consistent nominal trends point to potential matrix-dependent endocrine effects. Assessing paired urine and semen samples reduces exposure misclassification and provides novel insights into NEO distribution, highlighting the need for multi-matrix biomonitoring and larger longitudinal studies to clarify reproductive health risks.

## Data Availability

The original contributions presented in the study are included in the article/**Supplementary Material**. Further inquiries can be directed to the corresponding authors.

## Supplementary material

Figure S1 Representative chromatograms of Neonicotinoids (NEOs) and internal standards (ISs) in human samples. **(A)** Urine; **(B)** Semen.

Figure S2 Directed acyclic graph (DAG) for the association between NEO exposure and reproductive hormones levels. DAG was created using browser-based DAGitty v3.0 (http://www.dagitty.net/dags.html). Note: NEOs, Neonicotinoid Insecticide; BMI, body mass index.

Figure S3 Associations of NEOs and their metabolites in urine and semen with hormone levels. Abbreviations: FSH, follicle-stimulating hormone; E2, estradiol; PRL, prolactin; T, testosterone; LH, luteinizing hormone; T/LH, testosterone-to-LH ratio; DN, Desnitro-dinotefuran; UF, Dinotefuran-urea; Descynao-ACE, Descyano-acetamiprid; DM-ACE, Desmethyl-acetamiprid; DN-IMI-olefin, Desnitro-imidacloprid-olefin; DN-IMI, Desnitro-imidacloprid; IMI-urea, Imidacloprid-urea; 5-OH-IMI, 5-Hydroxy-imidacloprid; DNT, Dinotefuran; ACE, Acetamiprid; CLO, Clothianidin; THCP, Thiacloprid, IMI, Imidacloprid, IMIT, Imidaclothiz; CI, confidence interval; FDR, false discovery rate. Models were adjusted for age, BMI, season, smoking status, reproductive disease, occupation and education. Coefficients represent the change in reproductive hormone levels associated with a one-unit increase in ln-transformed NEO concentration.
